# Maximizing antimalarial efficacy and the importance of dosing strategies

**DOI:** 10.1186/s12916-015-0349-9

**Published:** 2015-05-09

**Authors:** James G Beeson, Philippe Boeuf, Freya JI Fowkes

**Affiliations:** Burnet Institute, 85 Commercial Road, Melbourne, Victoria 3004 Australia

**Keywords:** Amodiaquine, Artemisinins, Artesunate, Drug resistance, Malaria

## Abstract

Artemisinin-based combination therapies (ACTs) are the cornerstone for the treatment of malaria. However, confirmed resistance to artemisinins in South-East Asia, and reports of reduced efficacy of ACTs raise major concerns for malaria treatment and control. Without new drugs to replace artemisinins, it is essential to define dosing strategies that maximize therapeutic efficacy, limit the spread of resistance, and preserve the clinical value of ACTs. It is important to determine the extent to which reduced efficacy of ACTs reflects true resistance versus sub-optimal dosing, and quantify other factors that determine treatment failure. Pooled analyses of individual patient data from multiple clinical trials, by investigators in the Worldwide Antimalarial Resistance Network, have shown high overall efficacy for three widely used ACTs, artemether-lumefantrine, artesunate-amodiaquine, and dihydroartemisinin-piperaquine. Analyses also highlight that suboptimal dosing leads to increased risk of treatment failure, especially among children. In the most recent study, an analysis of clinical trials of artesunate-amodiaquine, widely used among children in Africa, revealed a superior efficacy for fixed-dose combination tablets compared to loose non-fixed dose combinations. This highlights the benefits of fixed-dose combinations as a practical strategy for ensuring optimal antimalarial dosing and maximizing efficacy.

Please see related article: http://www.biomedcentral.com/1741-7015/13/66

## Introduction

Artemisinin-based combination therapies (ACTs) have made a major contribution to the reduction in global malaria morbidity and mortality since their use became widespread approximately 10 years ago. ACTs are recommended by the WHO as the first-line treatment of uncomplicated and severe *P. falciparum* malaria in all areas in which malaria is endemic [1], and have been adopted as first-line therapy in many countries. Around 390 million ACT treatments are procured annually [2]. Drugs of the artemisinin group (artesunate, artemether, and dihydroartemisinin are the most used) are the most parasiticidal of established antimalarials and rapidly clear parasitemia, and are well tolerated with a good safety profile. However, artemisinin drugs have a short half-life (<1 hour) and when used on their own to treat malaria require 7- to 10-day treatment to achieve high cure rates, which impacts on adherence. Therefore, artemisinins are typically combined with a long-acting partner drug (e.g., lumefantrine, amodiaquine, piperaquine) in order to achieve high cure rates with a 3-day treatment regimen. The combination of artemether-lumefantrine is the most widely used ACT, and is highly efficacious [3]. Artesunate-amodiaquine (AS-AQ) is widely used for malaria therapy in Africa, particularly among children, and is the second most used ACT globally.

## The global threat of drug resistance

Unfortunately, the early signs of artemisinin resistance have emerged in South-East Asia threatening recent gains and milestones in the treatment and control of malaria [4,5]. Resistance to artemisinins has been recently associated with a mutation in the *kelch13* gene (gene ID PF3D7_1343700) [6-8], and the identification of this genetic marker will greatly facilitate resistance surveillance [4,9]. Emerging resistance was initially identified as delayed parasite clearance rates following treatment with artemisinin-based therapies [5]. Confirmed partial artemisinin resistance is now defined by the WHO as ≥5% of patients carrying K13 resistance-associated mutations, all of whom have been found, after treatment with ACT or artesunate monotherapy, to have either persistent parasitaemia by microscopy on day 3, or a parasite clearance half-life of ≥5 hours. Reflecting the importance of this issue, the WHO launched its Global Plan on Artemisinin Resistance Containment in 2011 with a specific emergency response to artemisinin resistance in the Greater Mekong sub-region in 2013. In addition, there are reports of reduced clinical efficacy of ACT therapy after 28 days of follow-up in some settings [10-15]. It is important to determine the extent to which this reduced efficacy reflects true resistance versus sub-optimal dosing, or other factors. The development of widespread resistance has limited the utility of numerous other antimalarials that were previously widely used, such as chloroquine and sulfadoxine-pyrimethamine, providing a sobering reminder of the potential impact of evolving resistance to drugs in current use. With no new drugs immediately available to replace artemisinins, it is essential to optimize and define dosing strategies to ensure maximum therapeutic efficacy of ACTs, limit the spread of resistance, and extend the clinical life of ACTs.

## Identifying factors associated with reduced efficacy: importance of dosing strategies

A new study from the Worldwide Antimalarial Resistance Network (WWARN) investigated risk factors for treatment failure with AS-AQ therapy [16]. Reduced antimalarial efficacy of AS-AQ has been reported in some studies, but it is unlikely that drug resistance is the major factor explaining this; confirmed AS resistance has not yet been reported in Africa [4,17] and reduced efficacy of AS-AQ has been observed between studies within the same region (which should have similar rates of potential AQ resistance) [18-20]. It was hypothesised that differences in doses or formulations impacted on the antimalarial efficacy of AS-AQ. To investigate this, the WWARN investigator group conducted a systematic review and meta-analysis of individual patient data including published and unpublished antimalarial therapeutic efficacy trials that included at least one AS-AQ arm, conducted between 1999 and 2012.

AS-AQ is available in three different body-weight-adjusted drug formulations: fixed-dose combinations, loose non-fixed dose combinations, and co-blistered non-fixed-dose combinations. All of these combinations aim at delivering 12 mg/kg of AS over 3 days, but the total target dose of AQ ranges from 25 to 30 mg/kg for loose non-fixed dose combinations and is 30 mg/kg for co-blistered non-fixed-dose combinations and fixed-dose combinations. The WWARN study included 43 trials (9,106 treatments), of which 39 trials (95% of subjects) were conducted in Africa, as well as 3 in Asia and 1 in South America, and the great majority were children (87.5% were <12 years old) [16]. The authors investigated the relationship between these different drug formulations and the actual dose of AQ received as well as treatment success, including parasitemia at 28 days and potential recrudescence in infants, children, and adults. Studies of this size are needed in order to have sufficient power to examine these questions when treatment efficacy rates are high, as they are with ACTs, emphasising the importance of multisite collaborative studies.

Arguably, the most important finding was that fixed-dose combinations were associated with the highest AS-AQ treatment efficacy in all age groups, including in children less than 5 years of age, independently of high baseline parasitemia and young age. The fixed dose combination of AS-AQ was developed using weight-for-age values from malaria endemic countries, to ensure optimal dosing [21,22]. Loose non-fixed-dose combinations, with an AQ target dose of 25 mg/kg, were associated with a 3.5-fold greater risk of recrudescence by day 28. The implication of these findings on the treatment of paediatric malaria reinforces initiatives to promote the distribution and implementation of fixed-dose regimens for therapy. The use of co-blistered and loose non-fixed-dose combinations may require splitting of tablets when treating children, potentially leading to suboptimal AQ dosing and therefore lower treatment efficacy. The use of fixed-dose AS-AQ formulations, including paediatric tablets, would circumvent this issue, leading to optimal AQ dosing and high treatment efficacy. As such, the treatment of uncomplicated *P. falciparum* malaria using fixed-dose AS-AQ formulations should be promoted for national treatment guidelines.

The study also found that the risk of recrudescence after AS-AQ treatment was higher among young children (<12 years old), those with a high baseline parasitemia, and in Asian studies compared to African. This may be reflective of the overall higher level of AQ resistance in Asia; however, given the small number of subjects from Asian trials, further studies are needed to examine this. The potentially crucial impact of *P. falciparum* resistance to AQ could not be evaluated in this study and further studies incorporating molecular markers of AQ resistance are warranted. Resistance to AQ is associated with mutations in the *pfcrt* and *pfmdr* genes [23,24], which are prevalent in most endemic countries [25]. It will be interesting to see the results from an AQ pharmacokinetic-pharmacodynamic analysis by WWARN, which is examining the effects of drug formulation and dose, and host age and nutritional status on AQ drug concentrations. Since most of the therapeutic efficacy trials analysed were performed in sub-Saharan Africa, more data from Asian and South American trials are needed in order to generalise these findings to different source populations with different risk factors and varied degrees of AQ resistance. Regarding side effects, there was no evidence for a higher risk of neutropenia (which is associated with AQ use) with the higher AQ dose, but higher rates of vomiting and diarrhoea were observed.

## Other studies investigating the importance of correct dosing with ACTs

There have been two related studies performed by WWARN investigators on the efficacy of other ACTs, highlighting the importance of optimal dosing. Dihydroartemisinin-piperaquine is another widely used ACT, and risk factors for recrudescence after treatment were examined in a pooled analysis of individual patient data from 26 efficacy studies (7,072 patients) [26]. Overall efficacy was high (97.7%), but was significantly reduced in those who received lower doses of piperaquine. Of concern was that 28.6% of young children (1 to 5 years old) received a piperaquine dose below the lower limit recommended by the WHO, further highlighting the need for strategies to ensure optimal dosing for malaria treatment. Recently, a pooled analysis of individual patient data from efficacy studies of artemether-lumefantrine included 61 studies and 14,327 patients [3]; overall, day 28 efficacy was very high (97.6%). Analysis revealed that a higher dose of artemether was associated with a lower risk of persistent parasitaemia on day 1 and lower gametocyte carriage rates, which may be important for reducing transmission. In Asia, lower doses of lumefantrine were associated with reduced efficacy among children weighing 10 to 15 kg. The risk of treatment failure was also higher among malnourished children aged 1 to 3 years in Africa.

## Conclusions

In an era of emerging artemisinin resistance, and the push for malaria elimination in many regions, studies such as that of the WWARN collaboration provide important data to inform policymakers and clinicians to optimise antimalarial therapies to maximize efficacy and help reduce the evolution of resistance (see Box 1 for a summary of key points). These findings provide further support for international collaborative networks and data sharing arrangements to address major challenges in global health, and the approach used for malaria has strong relevance to antimicrobial therapy and resistance more broadly. Individual patient meta-analyses of therapeutic efficacy studies are considered the strongest form of clinical evidence, and are essential to inform antimalarial policy and clinical treatment guidelines to ensure the rapid and effective treatment of malaria cases.

## Box 1: Key points

### Antimalarial drugs and resistance

- The WHO recommends artemisinin combination therapies (ACT) as the first-line treatment of malaria
- Around 390 million courses of ACTs procured annually ^1^
- Emerging resistance to ACTs: Resistance confirmed in five countries in the Greater Mekong sub-region
- Suspected artemisinin resistance is defined as failure to clear parasitemia within 3 days of commencing treatment, or a parasite clearance half-life of ≥5 hours
- Resistance to ACTs is associated with mutations in *kelch13* gene
- Resistance to other antimalarials is widespread (e.g. chloroquine, amodiaquine, sulfadoxine-pyrimethamine)
- Resistance to amodiaquine and chloroquine is associated with mutations in the *pfcrt and pfmdr1* genes
- Ensuring effective dosing is crucial to maximize efficacy and to protect against the further development and spread of resistance

### WWARN study on artesunate-amodiaquine (AS-AQ) ^2^

- AS-AQ is widely used in Africa, and is the second most widely used ACT
- AS-AQ Previously shown to have high efficacy when administered correctly
- WWARN study performed an individual patient meta-analysis using data from 43 studies (39 studies in Africa) with over 9,000 treatments, predominantly in children
- Study evaluated the impact of different dosing strategies on therapeutic efficacy
- Fixed-dose and co-blistered non-fixed dose combinations had high efficacy (98%)
- Use of loose tablets at non-fixed dose had significantly lower efficacy (up to 3.5-fold higher risk of treatment failure)
- Findings highlight the benefit of fixed dose combinations and support this strategy in the treatment and control of malaria
- Increased risk of recrudescence among young children, those with high baseline parasitemia, and among trials conducted in Asia

1. Based on WHO figures from 2013.
2. See reference [16]

## Abbreviations

ACTs: Artemisinin-based combination therapies
AS-AQ: Artesunate-amodiaquine
WWARN: Worldwide Antimalarial Resistance Network
